# Mediterranean diet is associated with better gastrointestinal health and quality of life, and less nutrient deficiency in children/adolescents with disabilities

**DOI:** 10.3389/fpubh.2023.1243513

**Published:** 2023-09-28

**Authors:** Hande Bakırhan, Volkan Özkaya, Merve Pehlivan

**Affiliations:** Department of Nutrition and Dietetics, Faculty of Health Sciences, Istanbul Medipol University, Istanbul, Türkiye

**Keywords:** children with disability, mediterranean diet, nutritional problems, nutritional deficiencies, gastrointestinal health, quality of life

## Abstract

**Background:**

Children and adolescents with disabilities face various nutritional problems. This study aimed to examine dietary characteristics, nutritional status and problems, gastrointestinal health, and quality of life in children and adolescents with disabilities.

**Methods:**

This study included 5–18 years old children and adolescents (*n* = 1,991) with disabilities. We used the Mediterranean Diet Quality Index (KIDMED), the Gastrointestinal Symptom Rating Scale (GSRS), and the Pediatric Quality of Life Inventory (PedsQL) to assess diet characteristics, gastrointestinal problems, and life quality. We collected retrospective 24-h food record to assess energy and nutrient intakes.

**Results:**

The rate of stunting in children with disabilities varies between 16.5% and 19.8%. When comparing disability types, more children with physical disabilities were underweight (8.8% vs. 6.7%) and stunted (19.8% vs. 16.5%), while more children with intellectual disabilities were tall (7.9% vs. 5.5%) and overweight/obese (21.1 vs. 17.2%; *p* < 0.05). Wasting (9.3%) and overweight/obesity (23.8%) were more common in children with disabilities aged 5–7 years (*p* < 0.001). Eating problems such as loss of appetite, food refusal, food neophobia, and food selectivity were more common in children aged 5–7 years, and problems with fast eating and overeating were more common in adolescents aged 13–18 years (*p* < 0.05). Among children and adolescents with disabilities, the nutrients with inadequate intakes were vitamin E, vitamin B1, folate, potassium, calcium, and iron, while the nutrients with intakes above the requirements were proteins, carbohydrates, vitamins A, B2, B6, B12, and C, phosphorus, zinc, and sodium. Participants with good Mediterranean diet quality had higher energy and nutrient intakes and higher percentages of meeting nutrient requirements (*p* < 0.05). KIDMED scores were negatively correlated with GSRS total (*r* = −0.14, *p* < 0.001) and subcomponent scores (abdominal pain, diarrhea, reflux, indigestion, and constipation; *p* < 0.05), and significantly and positively correlated with PedsQL total (*r* = 0.12, *p* < 0.001). A one-unit increase in the GSRS score resulted in a 14.4 times decrease in the PedsQL score, and a one-unit increase in the KIDMED score resulted in a 10.8 times increase in the PedsQL score (*p* = 0.001).

**Conclusion:**

Overweight/obesity, stunting/wasting, nutritional problems, and deficiencies are common among disabled children and adolescents. Mediterranean diet is associated with a better quality of life, and gastrointestinal health in children with disabilities.

## 1. Introduction

Persons with disabilities are individuals who have varying degrees of loss of physical, intellectual, psychological, sensory, and social abilities. They have difficulty adapting to social life and meeting their daily needs and require protective, nursing, rehabilitative, counseling and support services due to congenital or acquired factors. Chronic diseases, genetic diseases, accidents and trauma cause many children to become physically, intellectually, emotionally, and perceptually disabled (1–3). Worldwide, it is estimated that 93 million children (1 in 20) below the age of 15 have a moderate to severe disability (3). The UNICEF 2022 report states that approximately 240 million children and one in 10 children aged 0–17 years have some form of disability (4). Studies conducted in Turkey do not adequately contribute to the information about the disabled population. The rate of indicators related to disability in children aged 2–14 in Turkey is 1.1%–2.2% (5). However, the Turkey Child Survey 2022 reports that the rate of 5–17 years old children with functional difficulties in learning, communication, vision, hearing, and walking varies from 0.2% to 1.5% (6).

Cognitive, behavioral, or physical functioning deficits in children result in developmental delays and adversely affect the adaptive skills necessary to maintain daily life. Specifically, intellectual disability is characterized by deficits in physical, mental, adaptive, and social functioning during the developmental years of life (7, 8). Gastrointestinal symptoms such as aspiration, oral motor disorders, delayed gastric emptying, dysphagia, gastroesophageal reflux, and constipation, as well as neurodevelopmental problems, musculoskeletal disorders, and spasticity, are commonly reported in children with physical and neurological disabilities. In addition, the number, duration, and severity of comorbidities have been found to increase with the type and severity of disability (9–11). These conditions lead to feeding difficulties, food refusal and malnutrition, as well as overweight and obesity in children with disabilities (9, 12, 13). Malnutrition has been shown to be both a consequence and a cause of disability, and children with disabilities are known to be at high risk of malnutrition (13, 14). Studies report that malnutrition during the growing and developing years of a child with a disability reduces learning potential and increases the burden of disease (14–16). In addition, parental ratings of the physical and psychosocial status of children with physical impairments are different from those of the normative group (17), and the quality of life of children with severe developmental disorders is lower than that of their peers without disabilities (18). The Mediterranean diet is a balanced diet that provides most of the nutrients in optimal proportions. Mediterranean diet which reflects sustainable good diet quality may be a supportive factor for better mental health, because Mediterranean diet can improve the important outcomes of attention-deficit/hyperactivity disorder such as inflammation and oxidative stress, by including healthy food choices such as fish, fruits, vegetables, and whole grains (19). Adolescents with mental disabilities have been found to have a poor quality diet or a diet that needs improvement to adapt to Mediterranean dietary patterns (20). Since the Mediterranean diet is universally recommended as health-protective (21), it is thought to be associated with general well-being in children and adolescents with disabilities. Information on the relationship between dietary practices, nutritional status and problems, gastrointestinal problems, and quality of life among children and adolescents with disabilities is lacking in the literature. We conducted this study to investigate possible associations between dietary characteristics, nutritional status, gastrointestinal problems, and quality of life in disabled children and adolescents.

## 2. Materials and methods

### 2.1. Study design and sample selection

**Figure 1:**
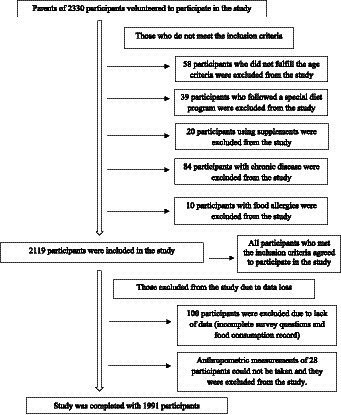
Participants recruitment flow chart.

This study was conducted between November and December 2022 on randomly selected 5–18 years old children and adolescents with various disabilities. In order to determine the sample size, the Turkey Child Survey 2022 disability rate was taken as a reference and the sample size was calculated as 1,991 children with 5% Type I error and 95% confidence interval (6). Special education and rehabilitation centers were contacted and invited to work. The study was conducted with 80 centers in 40 provinces that agreed to participate in the study. These children and adolescents were from different geographical regions of Turkey (seven regions, 40 cities), were diagnosed with developmental, sensory and/or physical disabilities by a medical board, and were attending special education and rehabilitation centers (*n* = 80). Parental consent was obtained for all children and adolescents included in this study. Children and adolescents with disabilities, chronic diseases (cardiovascular, renal, hepatic, diabetes, and cancer), special diets (ketogenic diet, low FODMAP, and elimination diet), nutritional support (enteral formula or tube feeding etc.), or food allergies/intolerances were excluded from the study (Figure 1). All participants continued to receive their routine individual therapy/training during the study. The Non-Interventional Clinical Research Ethics Committee of Istanbul Medipol University approved the study protocol with decision number 894 on 26 October 2022. Parents of all participants provided informed consent for this study that adhered the tenets of the Declaration of Helsinki.

### 2.2. Data collection

Information on sociodemographic characteristics, medical history, eating habits, eating problems, dietary characteristics, gastrointestinal problems, and quality of life of the children and adolescents included in the study was collected by administering questionnaires to mothers, fathers, or caregivers responsible for the care of the participant using face-to-face interview techniques.

### 2.3. Assessment of anthropometric measurements

Body weight and height measurements of the participants were taken by the personnel in the special education and rehabilitation centers. The personnel in the special education and rehabilitation centers were trained by the researchers in order to ensure standardization in body weight and height measurements. The instruments used to measure body weight and height were provided to centers by the researchers. Body weight was measured without shoes on a SECA-813 professional scale with an accuracy of 100 g. A Mesilife-13539 portable stadiometer with an accuracy of 1 mm was used to measure height. Participants’ heights were measured while standing upright and without shoes, with the Frankfort plane in the correct position. Knee length measurement was used to measure body height in children whose standing height could not be measured, who could not stand without support, and who were immobile. The measurement was taken with the child lying on his back, with the knee and ankle at a 90° angle, and a special caliper was used to measure the distance from the heel to the upper end of the patella. The measurements were taken with an accuracy of 0.1 cm, repeated three times for accuracy, and the average of the measurements was taken. Formulas based on age, gender, and disease condition were used to calculate the height of children using knee height. Stevenson formula was used for children aged 0–12 (22), and Chumlea formula was used for children over 12 years old (23). Anthropometric data were assessed using the WHO AnthroPlus Program and WHO Reference Values for Children 5–19 Years—2007 to calculate *z*-scores for weight-, height-, and body mass index (BMI)-for-age (24). These *z*-scores were considered normal if they were between −2SD and +2SD of the cut-off points. Scores below these cutoffs were considered to indicate that the participant was stunted/severely stunted, underweight/severely underweight, or wasting/severely wasting, and scores above these cutoffs were considered to indicate that the participant was very tall or overweight/obese (25).

### 2.4. Assessment of dietary characteristics

To determine the participants’ energy and nutrient intakes, researchers obtained a retrospective 24-h daily food record for each participant by asking relevant questions of the participant’s parent or caregiver. Energy and nutrient intakes were calculated using the food consumption information obtained and the full version of the Nutrition Information System 8.2 (BeBiS 8.2) software. Participants’ energy and nutrient intakes were assessed using the Dietary Reference Intake, which provides energy and nutrient requirements for age and gender (26). The Turkish version of the Mediterranean Diet Quality Index (KIDMED), designed by Serra-Majem et al. (27) and validated by Apaydin Kaya and Temiz (28), was used to assess the Mediterranean diet characteristics of the participants (27, 28). KIDMED is a 16-question index that assesses several dietary characteristics, including breakfast habits, frequency of consuming different foods, frequency of fast-food consumption, etc. It is widely used to assess compliance with the Mediterranean diet among children and adolescents (29). A KIDMED total score of ≥8 was considered a good quality Mediterranean diet, a score of 4–7 was considered an average quality Mediterranean diet, and a score of ≤3 was considered poor diet quality (28).

### 2.5. Assessment of gastrointestinal symptoms

The Gastrointestinal Symptom Rating Scale (GSRS) was designed by Revicki et al. (30). The validity and reliability study of the scale in the Turkish language was conducted by Turan et al. (31). The questionnaire asks how the person felt about gastrointestinal problems in the past week. The scale consists of 15 items grouped into five subdimensions, including abdominal pain [questions 1, 4, and 5 (abdominal pain, hunger pains, and nausea)], reflux [questions 2 and 3 (heartburn and acid reflux)], indigestion [questions 6, 7, 8, and 9 (stomach rumbling, abdominal bloating, burping, and increased gas passing or flatus)], diarrhea [questions 11, 12, and 14 (diarrhea, loose stools, and urgent need to defecate)], and constipation [questions 10, 13, and 15 (hard stools, constipation, and sensation of not completely emptying the bowels)]. Scale items are rated on a seven-point Likert scale ranging from “no discomfort at all” to “very severe discomfort. An item score of 1 indicates the absence of the symptom, while a score of 7 indicates frequent and severe discomfort. Higher scores on the scale indicate more severe symptoms (30, 31).

### 2.6. Pediatric quality of life inventory

The Pediatric Quality of Life Scale (PedsQL) was used to assess quality of life. Varni et al. (32) designed the scale to assess health-related quality of life in children and adolescents between the ages of 2 and 18 years. PedsQL is developed for specific age groups (young children: 5–7 years; children: 8–12 years; and adolescents: 13–18 years). Turkish validity and reliability of the PedsQL were made by Üneri et al. (33) for ages 5–7, Sönmez and Başbakkal for ages 8–12 (34), and Memik et al. (35) for ages 13–18 (33–35). The scale consists of subdimensions of physical, emotional, school, and social functioning. The PedsQL asks about physical well-being, emotional functioning, and social functioning, which are characteristics of health as defined by the World Health Organization (32, 36). The total score of the scale is calculated first. Second, the overall physical health score is calculated. Finally, the total score for psychosocial health is calculated as an assessment of emotional, school and social functioning. Items are scored from 0 to 100. A response of “never” is scored 100, “almost never” is scored 75, “sometimes” is scored 50, “often” is scored 25, and “almost always” is scored 0. Increased scores represent better health-related quality of life (37).

### 2.7. Data analysis

Parametric tests were used without performing a normality test because the data conformed to the central limit theorem (38). Data were analyzed using minimum and maximum values, median and mean and standard deviation (mean ± SD) for summarizing continuous variables. For summarizing categorical variables percentages (%) and frequencies (*n*) were used. Means of continuous values were compared between two independent groups using Student’s *t*-test. The one-way ANOVA test was used to compare the means of continuous values between more than two independent groups. A *post hoc* test (Tukey) was used for pairwise comparisons when a significant difference between groups was found. The relation between categorical variables was evaluated using Chi-squared test statistics. Pearson’s correlation test was used to assess the relationship between continuous variables. Since energy-adjusted intakes are important, we used energy-adjustment residual method. Energy adjusted multiple linear regression was also used in the data analysis. Since statistical significance was found between PedsQL and GSRS and KIDMED scores, energy intake, GSRS and KIDMED score values were included as independent variables and PedsQL score value was included as dependent variable in the regression. Values of *p* < 0.05 were accepted as statistically significant. Statistical analysis of the data was performed using IBM SPSS 21 statistical software package.

## 3. Results

This study included a total of 1,991 children and adolescents between the ages of 5 and 18, of whom 738 were physically disabled and 1,253 were intellectually disabled, 33.9% were between the ages of 5 and 7, 36.7% were between the ages of 8 and 12, and 29.4% were between the ages of 13 and 18. The distribution of the participants by gender and disability characteristics is shown in Table 1. While 62.9% of the participants were developmentally disabled, 37.1% were physically and emotionally disabled. While the most common type of disability among children and adolescents with physical and sensory disabilities is body movement restrictions (57.0%), the most common disability type in children and adolescents with developmental disabilities is autism spectrum disorder (38.5%; Table 1).

**Table 1:** Distribution of participants by gender and disability.

	Gender		
	*Female*	*Male*	Total (*n* = 1,991)
Disability	*n* (%)	*n* (%)	*n* (%)
**Physical and sensory**			
Restriction in body movement	219 (59.2)	202 (54.9)	421 (57.0)
Visual impairment	21 (5.7)	14 (3.8)	35 (4.7)
Hearing impairment	82 (22.1)	69 (18.8)	151 (20.5)
Speech and language impairment	48 (13.0)	83 (22.5)	131 (17.8)
*Total*	370 (50.1)	368 (49.9)	738 (37.1)
**Developmental**			
Learning and developmental disabilities	144 (24.9)	128 (19.0)	272 (21.7)
Intellectual disabilities	122 (21.1)	115 (17.0)	237 (18.9)
Internalizing disorder, mental illness, or mood disorder	132 (22.8)	130 (19.3)	261 (20.9)
Autism spectrum disorder	181 (31.2)	301 (44.7)	482 (38.5)
*Total*	579 (46.2)	674 (53.8)	1,253 (62.9)

^a^Participants’ physical, sensory, and developmental disabilities or problems were classified according to the disability categories defined in the studies of Turner et al. (39) and Jones et al. (40).

Table 2 shows participants’ gastrointestinal symptoms, eating problems, and quality of life by disability type, age group, and diet quality. Compared to participants with poor diet quality, ones with good diet quality had significantly higher total (1432.0 ± 403.56 vs. 1322.3 ± 395.25) and subcomponent scores on the quality of life scale and lower total (22.6 ± 8.74 vs. 25.8 ± 11.17, *p* < 0.001) and subcomponent scores on the GSRS (*p* < 0.05). However, participants with good diet quality had significantly lower the incidence of loss of appetite compared to other groups (*p* < 0.001). Food refusal (24.8% vs. 20.6%), fast eating (22.1% vs. 13.5%), overeating (17.3% vs. 8.8%), food neophobia (16.8% vs. 12.6%), food selectivity (35.4% vs. 27.8%), and crying spells while eating (8.8% vs. 6.0%) were significantly more frequent in the intellectually disabled, whereas difficulties with chewing (13.0% vs. 9.3%) and swallowing (12.2% vs. 7.2%) were significantly more frequent in the physically disabled (*p* < 0.05). Eating problems such as loss of appetite, food refusal, food neophobia, food selectivity, pocketing/pushing food out of mouth, and crying while eating were common in children with disabilities aged 5–7 years (*p* < 0.001, *p* < 0.001, *p* = 0.04, *p* = 0.003, *p* < 0.001, *p* < 0.001, and *p* = 0.009, respectively), and problems with fast eating and overeating were common in adolescents with disabilities aged 13–18 years (*p* < 0.001).

**Table 2:** Gastrointestinal symptoms, nutritional problems, and quality of life according to disability type, age group, and diet quality of the participants.

Gastrointestinal system health, quality of life, and nutritional problems		Disability			Age				KIDMED			
		*Physical and sensory*	*Developmental*	*p-*value	*5–7*	*8–12*	*13–18*	*p* value	*Poor*	*Average*	*Good*	*p-*value
		*Mean ± SD*	*Mean ± SD*		*Mean ± SD*	*Mean ± SD*	*Mean ± SD*		*Mean ± SD*	*Mean ± SD*	*Mean ± SD*	
		*(n = 738)*	*(n = 1,253)*		*(n = 675)*	*(n = 730)*	*(n = 586)*		*(n = 370)*	*(n = 1,023)*	*(n = 598)*	
GSRS	Total score	23.2 ± 9.31	23.8 ± 10.12	0.12	23.3 ± 9.51	23.6 ± 9.78	24.0 ± 10.26	0.40	25.8 ± 11.17	23.4 ± 9.79	22.6 ± 8.74	**<0.001**
	Abdominal pain	4.5 ± 2.31	4.5 ± 2.32	0.87	4.4 ± 2.24	4.6 ± 2.32	4.6 ± 2.41	0.24	5.1 ± 2.78	4.5 ± 2.31	4.2 ± 1.93	**<0.001**
	Reflux	2.6 ± 1.56	2.7 ± 1.82	0.09	2.5 ± 1.52	2.7 ± 1.81	2.7 ± 1.84	**0.02**	2.9 ± 1.91	2.7 ± 1.78	2.5 ± 1.51	**0.004**
	Diarrhea	4.2 ± 2.53	4.2 ± 2.41	0.91	4.2 ± 2.43	4.3 ± 2.59	4.2 ± 2.31	0.38	4.5 ± 2.78	4.2 ± 2.54	4.0 ± 2.06	**0.030**
	Indigestion	6.5 ± 3.49	6.9 ± 3.95	**0.006**	6.7 ± 3.78	6.7 ± 3.56	6.9 ± 4.07	0.42	7.3 ± 4.04	6.5 ± 3.47	6.8 ± 4.12	**0.004**
	Constipation	5.1 ± 3.71	5.3 ± 3.86	0.5	5.3 ± 3.81	5.1 ± 3.75	5.3 ± 3.92	0.59	5.9 ± 3.99	5.2 ± 3.96	4.8 ± 3.38	**<0.001**
PedsQL	Total score	1393.3 ± 393.06	1408.4 ± 398.31	0.42	1419.4 ± 369.19	1384.6 ± 404.47	1393.4 ± 409.31	0.34	1322.3 ± 395.25	1414.7 ± 389.04	1432.0 ± 403.56	**<0.001**
	Physical functioning	485.5 ± 207.41	498.6 ± 196.98	0.16	498.9 ± 189.22	483.1 ± 206.19	493.3 ± 207.84	0.42	468.9 ± 202.84	495.2 ± 199.33	506.8 ± 201.42	**0.02**
	Emotional functioning	305.5 ± 108.54	308.5 ± 109.05	0.54	307.3 ± 108.01	307.3 ± 107.69	307.8 ± 110.94	0.99	287.4 ± 106.49	310.8 ± 109.52	313.8 ± 107.86	**<0.001**
	Social functioning	305.8 ± 111.67	299.8 ± 111.29	0.25	305.8 ± 108.51	295.6 ± 110.31	297.8 ± 114.28	0.31	282.3 ± 106.91	305.3 ± 113.31	308.6 ± 109.75	**0.001**
	School functioning	311.2 ± 101.73	309.5 ± 105.68	0.72	315.1 ± 100.18	308.2 ± 103.41	306.2 ± 108.17	0.37	296.1 ± 105.85	313.1 ± 100.52	313.5 ± 10.76	**0.02**
		***n* (%)**			***n* (%)**				***n* (%)**			
Presence of nutritional problems	Loss of appetite	176 (23.8)	291 (23.2)	0.75	202 (29.9)	158 (21.6)	107 (18.3)	**<0.001**	104 (28.1)	259 (25.3)	104 (17.4)	**<0.001**
	Food refusal	152 (20.6)	311 (24.8)	**0.03**	201 (29.8)	164 (2.5)	98 (16.7)	**<0.001**	94 (25.4)	245 (23.9)	124 (20.7)	0.19
	Fast eating	100 (13.5)	277 (22.1)	**<0.001**	102 (15.1)	135 (18.5)	140 (23.9)	**<0.001**	70 (18.9)	195 (19.1)	112 (18.7)	0.99
	Overeating	65 (8.8)	217 (17.3)	**<0.001**	66 (9.8)	106 (14.5)	110 (18.8)	**<0.001**	48 (13)	139 (13.6)	95 (15.9)	0.34
	Food neophobia	93 (12.6)	211 (16.8)	**0.01**	120 (17.8)	111 (15.2)	73 (12.5)	**0.04**	56 (15.1)	164 (16)	84 (14)	0.56
	Food selectivity	205 (27.8)	443 (35.4)	**<0.001**	52 (37.3)	229 (31.4)	167 (28.5)	**0.003**	123 (33.2)	338 (33)	187 (31.3)	0.73
	Painful swallowing	29 (3.9)	34 (2.7)	0.14	21 (3.1)	21 (2.9)	21 (3.6)	0.76	11 (3)	37 (3.6)	15 (2.5)	0.46
	Difficulty in chewing	96 (13.0)	117 (9.3)	**0.01**	85 (12.6)	70 (9.6)	58 (9.9)	0.14	46 (12.4)	110 (10.8)	57 (9.5)	0.36
	Difficulty in swallowing	90 (12.2)	90 (7.2)	**<0.001**	66 (9.8)	59 (8.1)	55 (9.4)	0.51	43 (11.6)	89 (8.7)	48 (8)	0.14
	Taking food out of the mouth	85 (11.5)	154 (12.3)	0.61	113 (16.7)	71 (9.7)	55 (9.4)	**<0.001**	54 (14.6)	125 (12.2)	60 (10)	0.10
	Storing food in the mouth	95 (12.9)	153 (12.2)	0.67	113 (16.7)	82 (11.2)	53 (9)	**<0.001**	47 (12.7)	137 (13.4)	64 (10.7)	0.28
	Crying while eating	44 (6.0)	110 (8.8)	**0.02**	67 (9.9)	56 (7.7)	31 (5.3)	**0.009**	35 (9.5)	77 (7.5)	42 (7)	0.36
	Choking while eating	47 (6.4)	69 (5.5)	0.43	37 (5.5)	33 (4.5)	46 (7.8)	**0.03**	20 (5.4)	60 (5.9)	36 (6)	0.92

Student’s *t* Chi-Square, One Way ANOVA-*post hoc* test-Tukey. Bold values are statistically significant (*p* < 0.05).

Table 3 shows the body composition of the participants by disability type, age group, and diet quality. The intellectually disabled participants had significantly higher mean body weight, height, and BMI of than those of the physically disabled participants (*p* < 0.05). Rates of wasting (9.3%) and overweight/obesity (23.8%) were higher in the 5–7 years old age group than those in other age groups (*p* < 0.001). Stunting rates ranged from 16.5 to 19.8%. When comparing participants with intellectual and physical disabilities, more children with physical disabilities were underweight (8.8% vs. 6.7%) and stunted (19.8% vs. 16.5%), whereas more children and adolescents with intellectual disabilities were tall (>+ 2SD, 7.9 vs. 5.5%) and overweight/obese (21.1 vs. 17.2%; *p* < 0.05).

**Table 3:** Anthropometric measurements of the participants according to disability type, age group and diet quality.

Anthropometry		Disability			Age				KIDMED			
		*Physical and sensory*	*Developmental*	*p-*value	*5–7*	*8–12*	*13–18*	*p* value	*Poor*	*Average*	*Good*	*p-*value
		*Mean ± SD (n = 738)*	*Mean ± SD (n = 1,253)*		*Mean ± SD (n = 675)*	*Mean ± SD (n = 730)*	*Mean ± SD (n = 586)*		*Mean ± SD (n = 370)*	*Mean ± SD (n = 1,023)*	*Mean ± SD (n = 598)*	
Anthropometry	Body mass index (*z* score)	0.44 ± 1.94	0.71 ± 1.81	**0.002**	0.65 ± 2.27	0.79 ± 1.72	0.39 ± 1.64	**0.001**	0.62 ± 1.85	0.61 ± 1.85	0.58 ± 1.89	0.96
	Height (*z* score)	−0.71 ± 1.87	−0.43 ± 1.76	**0.002**	−0.51 ± 1.97	−0.48 ± 1.81	−0.77 ± 1.66	**0.01**	−0.45 ± 1.82	−0.62 ± 1.78	−0.44 ± 1.84	0.12
	Weight (*z* score)	0.11 ± 1.59	0.42 ± 162	**0.002**	0.15 ± 1.61	0.31 ± 1.53	4.58 ± 2.17	**<0.001**	0.26 ± 1.63	0.24 ± 1.59	0.43 ± 1.64	0.26
	Weight (kg)	34.6 ± 16.88	38.8 ± 18.61	**<0.001**	21.1 ± 5.34	34.5 ± 10.96	56.2 ± 17.02	**<0.001**	36.2 ± 18.94	36.9 ± 17.69	38.5 ± 18.22	0.12
	Body mass index (kg/m^2^)	18.8 ± 6.21	19.7 ± 5.08	**<0.001**	17.1 ± 6.69	19.1 ± 4.41	22.2 ± 5.19	**<0.001**	19.5 ± 7.73	19.3 ± 4.86	19.5 ± 5.01	0.85
		***n* (%)**			***n* (%)**				***n* (%)**			
Height classification (*z* score)	<−3 SD	76 (10.3)	75 (6)	**0.008**	47 (7)	62 (8.5)	42 (7.2)	**<0.001**	25 (6.8)	82 (8.0)	44 (7.4)	0.62
	−3 SD - −2 SD	70 (9.5)	131 (10.5)		67 (9.9)	69 (9.5)	65 (11.1)		36 (9.7)	107 (10.5)	58 (9.7)	
	−2 SD - 0 SD	350 (47.4)	599 (47.8)		298 (44.1)	336 (46)	315 (53.8)		173 (46.8)	506 (49.5)	270 (45.2)	
	0 SD - +2 SD	201 (27.2)	350 (27.9)		194 (28.7)	213 (29.2)	144 (24.6)		105 (28.4)	262 (25.6)	184 (30.8)	
	+2 SD - +3SD	21 (2.8)	56 (4.5)		38 (5.6)	31 (4.2)	8 (1.4)		16 (4.3)	36 (3.5)	25 (4.2)	
	>+ 3 SD	20 (2.7)	42 (3.4)		31 (4.6)	19 (2.6)	12 (2.0)		15 (4.1)	30 (2.9)	17 (2.8)	
Body mass index classification (*z* score)	<−3 SD	32 (4.3)	36 (2.9)	**0.04**	36 (5.3)	18 (2.5)	14 (2.4)	**<0.001**	15 (4.1)	28 (2.7)	25 (4.2)	0.12
	−3 SD - −2 SD	33 (4.5)	48 (3.8)		27 (4.0)	28 (3.8)	26 (4.4)		11 (3)	52 (5.1)	18 (3)	
	−2 SD - 0 SD	213 (28.9)	314 (25.1)		195 (28.9)	159 (21.8)	173 (29.5)		95 (25.7)	282 (27.6)	150 (25.1)	
	0 SD - +2 SD	333 (45.1)	590 (47.1)		256 (37.9)	380 (52.1)	287 (49)		180 (48.6)	448 (43.8)	295 (49.3)	
	+2 SD - +3SD	79 (10.7)	182 (14.5)		84 (12.4)	107 (14.7)	70 (11.9)		41 (11.1)	145 (14.2)	75 (12.5)	
	>+ 3 SD	48 (6.5)	83 (6.6)		77 (11.4)	38 (5.2)	16 (2.7)		28 (7.6)	68 (6.6)	35 (5.9)	

Student’s *t* Chi-Square, One Way ANOVA-*post hoc* test-Tukey. Bold values are statistically significant (*p* < 0.05).

Table 4 shows energy and nutrient intakes and the percentage of participants meeting nutrient intakes requirements by disability type, age group, and diet quality. Disabled children and adolescents with good diet quality had higher energy (1364.1 ± 455.65 vs. 1254.5 ± 455.88 kcal and 1229.8 ± 492.59 kcal) and nutrient intakes, and their percentages of meeting required nutrient intakes (protein, vitamin A, B1, B2, B6, B12, C, and E, folate, potassium, sodium, calcium, magnesium, phosphorus, iron, and zinc) were statistically significantly higher (*p* < 0.05). Nutrients with inadequate intake in children and adolescents with disabilities were vitamin E (85.7% and 91.7%, vitamin E intake was adequate only in those with good diet quality), vitamin B1 (83.0%, 84.5%, and 92.8%), folate (70.0%, 75.5%, and 89%), potassium (40.7%, 43.6%, and 50.3%), calcium (51.4%, 55.4%, and 66.0%), and iron (74.6%, 75.2%, and 81.4%). The nutrients with more than adequate intakes in this patient population of children and adolescents with disabilities were protein (168.2%, 177.4%, and 193.2%), carbohydrate (108.2%, 108.9%, and 112.5%), vitamin B2 (134.1%, 145.1%, and 169.9%), vitamin B6 (109.6%, 115.3%, and 128.4%), vitamin B12 (211.3%, 222.4%, and 253.9%), phosphorus (106.9%, 110.9%, and 125.8%), and sodium (174.9%, 171.0%, and 202.3%). When the data were analyzed according to the type of disability, the percentages of children and adolescents with physical and sensory disabilities meeting the required intakes of protein, calcium, phosphorus, and zinc were significantly higher than those with intellectual disabilities (*p* = 0.004, *p* = 0.03, *p* = 0.01, and *p* = 0.04, respectively). Table 5 shows the energy adjusted nutrients intakes of the participants. When the dietary intakes of disabled individuals are examined; it was determined that they consumed 142.8 ± 33.40 g carbohydrate, 51.6 ± 13.47 g protein, and 56.1 ± 13.53 g fat.

**Table 4:** Energy and nutrients intakes of participants according to disability type, age groups, and diet quality.

*Energy and nutrients intakes and meeting dietary requirements (%)*	Disability			Age				KIDMED			
	*Physical and sensory (Mean ± SD) (n = 738)*	*Developmental (Mean ± SD) (n = 1,253)*	*p-*value	*5–7 (Mean ± SD) (n = 675)*	*8–12 (Mean ± SD) (n = 730)*	*13–18 (Mean ± SD) (n = 586)*	*p-*value	*Poor (Mean ± SD) (n = 370)*	*Average (Mean ± SD) (n = 1,023)*	*Good (Mean ± SD) (n = 598)*	*p-*value
Energy (kcal)	1271.9 ± 375.59	1289.2 ± 459.94	0.42	1208.4 ± 453.11	1285.6 ± 449.55	1355.7 ± 489.25	**<0.001**	1229.8 ± 492.59	1254.5 ± 455.88	1364.1 ± 455.65	**<0.001**
Protein (g)	51.2 ± 21.75	51.8 ± 21.11	0.51	49.2 ± 20.82	51.7 ± 20.51	53.9 ± 22.01	**0.002**	46.8 ± 21.35	50.2 ± 20.2	56.9 ± 22.16	**<0.001**
Carbohydrate (g)	140.4 ± 62.94	144.2 ± 64.43	0.19	135.2 ± 67.22	144.9 ± 61.58	149.5 ± 63.83	**0.001**	140.7 ± 69.74	141.6 ± 64.14	146.2 ± 59.52	0.28
Total fat (g)	56.1 ± 24.43	56.0 ± 24.22	0.95	52.2 ± 21.51	55.4 ± 23.81	60.2 ± 27.03	**<0.001**	53.2 ± 24.66	54.1 ± 23.552	61.2 ± 24.61	**<0.001**
Saturated fat (g)	22.9 ± 11.54	22.7 ± 10.74	0.78	21.5 ± 10.52	22.6 ± 10.61	24.2 ± 11.83	**<0.001**	21.3 ± 10.71	21.8 ± 10.81	25.3 ± 11.22	**<0.001**
Cholesterol (mg)	280.0 ± 169.98	275.4 ± 169.72	0.56	271.5 ± 11.12	269.3 ± 162.82	288.5 ± 187.23	0.11	239.3 ± 164.56	273.4 ± 169.42	307.0 ± 168.56	**<0.001**
Vitamin A (mg)	778.8 ± 628.66	805.8 ± 651.35	0.36	752.0 ± 511.97	806.7 ± 691.05	838.0 ± 594.83	0.12	681.5 ± 540.18	763.0 ± 680.34	922.6 ± 515.97	**<0.001**
Vitamin C (mg)	68.0 ± 44.97	72.6 ± 51.34	**0.04**	66.8 ± 39.78	71.7 ± 39.92	74.1 ± 39.51	**0.05**	61.5 ± 38.12	67.7 ± 36.51	82.3 ± 22.05	**<0.001**
Vitamin E (mg)	8.7 ± 5.77	8.9 ± 5.52	0.57	8.2 ± 5.28	8.7 ± 5.42	9.6 ± 6.19	**<0.001**	7.8 ± 5.16	8.6 ± 4.48	9.9 ± 4.62	**<0.001**
Vitamin B1 (mg)	0.7 ± 0.71	0.8 ± 0.65	0.12	0.7 ± 0.33	0.7 ± 0.45	0.8 ± 0.62	0.13	0.6 ± 0.48	0.7 ± 0.67	0.8 ± 0.65	0.13
Vitamin B2 (mg)	1.2 ± 0.63	1.1 ± 0.75	0.57	1.1 ± 0.92	1.1 ± 0.56	1.2 ± 0.74	0.18	1.0 ± 0.48	1.1 ± 0.65	1.3 ± 0.88	**<0.001**
Vitamin B6 (mg)	0.9 ± 0.46	0.9 ± 0.47	0.26	0.9 ± 0.54	0.9 ± 0.43	1.0 ± 0.45	**0.004**	0.8 ± 0.45	0.9 ± 0.42	1.0 ± 0.52	**<0.001**
Vitamin B12 (mcg)	3.6 ± 2.25	3.6 ± 2.33	0.97	3.5 ± 1.07	3.5 ± 2.36	3.7 ± 242	0.16	3.1 ± 1.78	3.5 ± 1.45	4.0 ± 2.14	**<0.001**
Folate (mcg)	205.7 ± 98.18	208.5 ± 97.75	0.53	19.4 ± 93.84	210.6 ± 94.35	221.5 ± 104.71	**<0.001**	179.3 ± 91.56	199.2 ± 82.33	239.0 ± 92.78	**<0.001**
Sodium (mg)	2376.9 ± 1785.26	2407.2 ± 1639.45	0.7	2214.6 ± 1167.11	2385.5 ± 1162.74	2527.1 ± 1812.45	**0.004**	2217.3 ± 1138.58	2329.3 ± 1704.91	2620.6 ± 1921.91	**<0.001**
Potassium (mg)	1900.8 ± 887.84	1906.1 ± 717.41	0.88	1819.2 ± 924.22	1912.8 ± 717.26	1997.6 ± 770.97	**0.002**	1703.8 ± 670.71	1843.2 ± 683.95	2132.3 ± 765.42	**<0.001**
Calcium (mg)	682.2 ± 373.34	660.6 ± 306.31	0.16	661.1 ± 384.32	655.5 ± 311.16	695.8 ± 321.66	0.09	584.7 ± 274.93	641.3 ± 295.09	767.1 ± 343.31	**<0.001**
Phosphorus (mg)	887.4 ± 361.36	890.0 ± 369.45	0.88	846.5 ± 348.45	896.2 ± 387.21	926.1 ± 365.02	**0.002**	800.2 ± 334.31	858.7 ± 354.04	995.8 ± 381.98	**<0.001**
Magnesium (mg)	195.1 ± 86.29	199.0 ± 98.01	0.37	184.9 ± 120.51	199.0 ± 80.46	209.6 ± 89.64	**<0.001**	187.2 ± 97.66	189.6 ± 74.82	217.5 ± 86.49	**<0.001**
Iron (mg)	7.3 ± 4.45	7.4 ± 3.37	0.81	6.9 ± 4.01	7.5 ± 3.44	7.8 ± 4.15	**<0.001**	7.1 ± 4.41	7.2 ± 3.68	7.8 ± 3.71	**0.003**
Zinc (mg)	8.0 ± 5.47	7.8 ± 3.39	0.38	7.5 ± 3.95	7.7 ± 3.72	8.5 ± 6.03	**0.001**	7.3 ± 3.12	7.5 ± 3.59	8.8 ± 4.59	**<0.001**
**Meeting dietary requirements (%)** ^*^											
Protein	189.6 ± 81.65	175.0 ± 98.21	**0.004**	250.6 ± 126.17	165.2 ± 86.42	119.2 ± 60.33	**<0.001**	168.2 ± 98.31	177.4 ± 96.03	193.2 ± 95.53	**0.001**
Carbohydrate	108.0 ± 48.41	110.9 ± 49.56	0.19	105.6 ± 50.67	109.7 ± 4.41	115.0 ± 49.11	**0.003**	108.2 ± 53.65	108.9 ± 49.34	112.5 ± 45.78	0.28
Vitamin A	152.6 ± 92.59	151.4 ± 98.86	0.84	189.7 ± 46.45	147.4 ± 95.05	114.0 ± 85.54	**<0.001**	136.4 ± 98.63	145.1 ± 98.04	173.1 ± 83.86	**<0.001**
Vitamin C	189.9 ± 94.53	192.5 ± 87.22	0.71	263.5 ± 152.75	183.4 ± 98.22	119.7 ± 65.56	**<0.001**	176.7 ± 96.43	183.8 ± 82.35	214.7 ± 112.45	**<0.001**
Vitamin E	94.8 ± 57.27	92.8 ± 52.53	0.45	119.5 ± 74.83	89.1 ± 47.27	69.7 ± 36.34	**<0.001**	85.7 ± 41.75	91.7 ± 54.62	101.8 ± 53.21	**<0.001**
Vitamin B1	88.3 ± 35.56	85.8 ± 32.66	0.23	105.2 ± 47.23	84.1 ± 30.25	68.7 ± 34.56	**<0.001**	83.0 ± 38.21	84.5 ± 23.24	92.8 ± 31.25	**<0.001**
Vitamin B2	155.8 ± 80.07	147.3 ± 73.63	**0.02**	191.5 ± 63.26	141.3 ± 55.27	114.7 ± 55.86	**<0.001**	134.1 ± 63.14	145.1 ± 60.26	169.9 ± 73.61	**<0.001**
Vitamin B6	120.1 ± 68.19	117.1 ± 64.95	0.32	153.7 ± 62.79	110.6 ± 59.3	86.8 ± 34.51	**<0.001**	109.6 ± 59.61	115.3 ± 53.99	128.4 ± 56.51	**<0.001**
Folate	80.0 ± 43.86	77.8 ± 40.57	0.25	97.0 ± 47.48	77.2 ± 3.31	59.3 ± 28.59	**<0.001**	70.0 ± 31.34	75.5 ± 39.33	89.3 ± 44.16	**<0.001**
Vitamin B12	237.4 ± 126.53	225.4 ± 113.25	0.09	297.3 ± 153.46	216.9 ± 124.96	167.9 ± 91.69	**<0.001**	211.3 ± 91.31	222.4 ± 98.93	253.9 ± 114.42	**<0.001**
Sodium	181.1 ± 86.69	181.2 ± 90.63	0.99	198.5 ± 90.57	170.9 ± 86.62	174.0 ± 95.78	**0.01**	174.9 ± 99.84	171.0 ± 56.45	202.3 ± 120.25	**0.004**
Potassium	45.4 ± 22.21	44.9 ± 17.26	0.52	48.4 ± 22.75	43.8 ± 17.21	42.9 ± 16.59	**<0.001**	40.7 ± 24.82	43.6 ± 16.43	50.3 ± 18.66	**<0.001**
Calcium	59.9 ± 34.93	56.6 ± 27.21	**0.03**	66.6 ± 36.61	53.3 ± 25.93	53.5 ± 24.73	**<0.001**	51.4 ± 22.36	55.4 ± 26.16	66.0 ± 30.76	**<0.001**
Phosphorus	120.1 ± 63.82	111.4 ± 59.03	**0.01**	171.5 ± 69.25	94.7 ± 42.55	74.1 ± 29.18	**<0.001**	106.9 ± 69.82	110.9 ± 67.56	125.8 ± 75.94	**<0.001**
Magnesium	105.6 ± 61.87	101.7 ± 62.44	0.22	145.5 ± 84.55	97.1 ± 40.25	61.8 ± 29.36	**<0.001**	102.8 ± 64.18	98.8 ± 5.51	110.9 ± 62.07	**0.003**
Iron	75.8 ± 44.78	77.6 ± 38.96	0.33	70.2 ± 38.51	88.8 ± 42.09	70.0 ± 39.83	**<0.001**	74.6 ± 44.11	75.2 ± 39.31	81.4 ± 42.24	**0.006**
Zinc	123.4 ± 72.71	115.9 ± 56.09	**0.04**	152.9 ± 76.92	109.2 ± 61.44	91.1 ± 45.33	**<0.001**	112.9 ± 54.57	114.1 ± 65.81	130.1 ± 65.02	**<0.001**

Student’s *t* Chi-Square, One Way ANOVA-*post hoc* test-Tukey, ^*^Nutrient intakes were assessed using the Dietary Reference Intake, which provides energy and nutrient requirements for age and gender. Bold values are statistically significant (*p* < 0.05).

**Table 5:** Energy adjusted nutrients intakes of the participants.

Energy adjusted nutrients	Dietary intake (Mean ± SD; *n* = 1,991)
Carbohydrate (g)	142.8 ± 33.40
Protein (g)	51.6 ± 13.47
Total fat (g)	56.1 ± 13.53
Cholesterol (mg)	277.0 ± 156.06
Fiber (g)	13.3 ± 5.76
Vitamin A (mg)	795.8 ± 617.01
Vitamin C (mg)	77.9 ± 35.73
Vitamin E (mg)	8.8 ± 4.96
Vitamin B1 (mg)	221.9 ± 127.27
Vitamin B2 (mg)	87.3 ± 47.28
Vitamin B6 (mg)	147.8 ± 61.61
Vitamin B12 (mcg)	3.6 ± 1.99
Folate (mcg)	118.4 ± 59.06
Sodium (mg)	192.6 ± 146.90
Potassium (mg)	1904.2 ± 606.64
Calcium (mg)	668.6 ± 286.52
Phosphorus (mg)	889.1 ± 254.99
Magnesium (mg)	197.6 ± 70.68
Iron (mg)	7.4 ± 3.04
Zinc (mg)	7.9 ± 3.81

Energy-adjustment residual method was used.

Table 6 shows the relationship of energy and nutrient intake to quality of life, gastrointestinal health, and body composition. Consumption of energy, carbohydrates, saturated fat, calcium, and potassium was associated with better quality of life (*p* < 0.05), and consumption of total fat, saturated fat, calcium, and vitamin B12 was associated with more severe gastrointestinal symptoms (*p* < 0.05). Nutrients other than carbohydrates and calcium were positively correlated with height *z*-score at a low level (*p* < 0.05).

**Table 6:** The relationship between energy and nutrients intake and quality of life, gastrointestinal system health, and body composition.

Energy and nutrients intakes (*n* = 1,991)	PedsQL score		GSRS score		Height *z* score		Weight *z* score	
	*r*	*p-*value	*r*	*p-*value	*r*	*p-*value	*r*	*p-*value
Energy (kcal)	0.05	**0.02**	0.03	0.20	0.06	**0.01**	0.03	0.36
Protein (g)	0.03	0.14	−0.01	0.65	0.06	**0.01**	0.03	0.30
Carbohydrate (g)	0.05	**0.01**	0.01	0.54	0.04	0.07	0.03	0.41
Total fat (g)	0.03	0.13	0.05	**0.03**	0.05	**0.02**	0.02	0.57
Saturated fat (g)	0.05	**0.04**	0.06	**0.01**	0.05	**0.03**	−0.03	0.37
Vitamin A (mg)	0.03	0.21	−0.02	0.49	0.07	**0.003**	0.03	0.43
Vitamin C (mg)	0.04	0.08	−0.02	0.48	0.06	**0.009**	0.05	0.09
Vitamin E (mg)	0.02	0.48	0.003	0.9	0.07	**0.003**	0.02	0.48
Vitamin B1 (mg)	0.05	**0.03**	0.04	0.06	0.05	**0.05**	0.004	0.91
Vitamin B2 (mg)	−0.009	0.73	0.001	0.96	0.06	**0.01**	−0.05	0.11
Vitamin B6 (mg)	0.04	0.07	−0.03	0.27	0.07	**0.002**	0.02	0.61
Vitamin B12 (mcg)	0.009	0.69	0.05	**0.02**	0.05	**0.02**	−0.001	0.96
Folate (mcg)	0.02	0.29	−0.03	0.18	0.07	**0.003**	0.07	**0.02**
Sodium (mg)	0.04	0.09	−0.02	0.48	0.02	0.29	0.04	0.23
Potasium (mg)	0.05	**0.02**	−0.002	0.91	0.05	**0.04**	0.04	0.26
Calcium (mg)	0.05	**0.03**	0.06	**0.01**	0.04	0.10	−0.004	0.91
Phosphorus (mg)	0.03	0.14	0.02	0.35	0.06	**0.008**	−0.001	0.97
Magnesium (mg)	0.03	0.14	0.009	0.70	0.06	**0.006**	0.04	0.17
Iron (mg)	−0.008	0.72	−0.003	0.91	0.02	0.29	0.01	0.65
Zinc (mg)	−0.008	0.71	0.01	0.57	0.06	**0.004**	−0.01	0.71

Pearson’s correlation test. Bold values are statistically significant (*p* < 0.05).

Table 7 shows the relation between the characteristics of the Mediterranean diet and quality of life, gastrointestinal health, and anthropometric measurements. While KIDMED scores and total (*r* = 0.12, *p* < 0.001) and subcomponent scores (respectively, *r* = 0.08, *p* < 0.001; *r* = 0.09, *p* < 0.001; *r* = 0.10, *p* < 0.001; and *r* = 0.07, *p* = 0.004) of the quality of life scale showed a low-level but significant and positive correlation, there was a negative low-level and significant correlation between KIDMED scores and total (*r* = −0.14, *p* < 0.001) and subcomponent scores of the GSRS (reflux, abdominal pain, indigestion, diarrhea, and constipation; respectively, *r* = −0.15, *p* < 0.001; *r* = −0.10, *p* < 0.001; *r* = −0.08, *p* = 0.001; *r* = −0.06, *p* = 0.008; and *r* = −0.12, *p* < 0.001). Among the anthropometric measures, the diet quality score was positively and significantly correlated only with mean body weight (*p* < 0.05). It was determined that a one-unit increase in the GSRS score resulted in a 14.4 times (95% CI, −16.03 to 12.75, *p* = 0.001) decrease in the PedsQL score, and a one-unit increase in the KIDMED score resulted in an increase of 10.8 times (95% CI, 4.84–16.81, *p* = 0.001) in the PedsQL score (not shown in the table).

**Table 7:** Relationship between Mediterranean diet characteristics and quality of life, gastrointestinal system health, and anthropometric measurements.

Quality of life, gastrointestinal system health, and anthropometric measurements (*n* = 1,991)		KIDMED	
		*r*	*p-*value
PedsQL	Total score	0.12	**<0.001**
	Physical functioning	0.08	**<0.001**
	Emotional functioning	0.09	**<0.001**
	Social functioning	0.10	**<0.001**
	School functioning	0.07	**0.004**
GSRS	Total score	−0.14	**<0.001**
	Abdominal pain	−0.15	**<0.001**
	Reflux	−0.10	**<0.001**
	Diarrhea	−0.08	**0.001**
	Indigestion	−0.06	**0.008**
	Constipation	−0.12	**<0.001**
Height (*z* score)		0.02	0.40
Weight (*z* score)		0.03	0.35
Body mass index (*z* score)		−0.01	0.53
Weight (kg)		0.05	**0.02**
Body mass index (kg/m^2^)		0.004	0.87

Pearson’s correlation test. Bold values are statistically significant (*p* < 0.05).

## 4. Discussion

The close link between diet and disability is established: malnutrition can contribute to or directly cause disability, and disability can lead to malnutrition. Infants and children are at high risk because malnutrition in early life has long-term consequences. Therefore, addressing nutritional problems is necessary (14). It is known that children and adolescents with severe disabilities are at a higher risk of malnutrition than their peers (41). One study in the literature reported that disabled children are three times more prone to be low weight and more prone be malnourished than those without disabilities (12). Another study reported that disabled children were 6.6 times more prone to be severely low weight and 11.8 times more prone to be severely stunted compared to controls of the same age and gender (9). Results from a systematic review study showed that compared to controls, children with all types of impairments were almost three times more prone to be low weight (OR 2.97, 95% CI) and almost twice as likely to be stunted and wasted (stunting: 1.82, wasting: 1.90) (12). A meta-analysis study reported that adolescents with intellectual disabilities had a 1.54 times higher risk of being overweight and a 1.80 times higher risk of obesity compared to those without disabilities (42). A systematic review study evaluating the prevalence of health problems among people with intellectual disabilities reported that the prevalence of obesity/overweight ranged from 3.9% to 34.8% (43). A study (*n* = 527) of 2–19 years old children and adolescents with physical and sensory disabilities reported that 18.8% of participants were overweight and 17.8% were obese, and the prevalence of obesity and stunting was high at 24.5% (44). The present study showed that the prevalence of obesity (14.6%–23.8%) and underweight (6.3%–9.3%) in children and adolescents with disabilities is similar in different age groups. The rates of wasting (9.3%) and obesity (23.8%), especially among 5–7 years old children with disabilities, are higher than the rates seen in other age groups (*p* < 0.001). However, the rate of stunting in our study varies from 16.5 to 19.8%; more children with physical disabilities are underweight compared to children with intellectual disabilities (8.8% vs. 6.7%), and more children and adolescents with intellectual disabilities are obese (21.1% vs. 17.2%; *p* = 0.04). A cross-sectional study (*n* = 345) of children with disabilities reported that 45.0% of participants were stunted, 19.0% were underweight, and 12.0% were overweight. This study also reported that intellectual disabled children were significantly more prone to be overweight or obese than their counterparts with sensory disabilities (OR = 5.84, 95% CI) (45). Given the information in the literature, it can be argued that different types and severities of disability are associated with different types of malnutrition (41, 45). Obesity and stunting are common, and information in the literature suggests that they may be caused by nutritional problems that negatively affect the normal growth and development of disabled children and adolescents. Difficulties in chewing and swallowing, eating problems such as food refusal, obsession with eating certain types of food, or food selectivity, low or high socioeconomic levels can lead to inadequate or excessive energy intake (44). In this study, we observed various feeding problems, including food refusal, food selectivity, loss of appetite, overeating, difficulty swallowing and chewing, and inadequate nutrient intake in children and adolescents with disabilities. Eating problems, including loss of appetite, food refusal, food neophobia, food selectivity, pocketing/pushing food out of mouth, and crying spells while eating, were common in children with disabilities aged 5–7 years, and problems with fast eating and overeating were common in adolescents with disabilities aged 13–18 years (*p* < 0.05). Risk factors for malnutrition and disability are complex and include a variety of biological, physical, environmental, and social factors. There is a need for comprehensive studies that examine the relevant risks in order to address the negative consequences associated with nutritional problems experienced by people with disabilities (14).

People with intellectual disabilities suffer from a variety of nutritional problems, including difficulty maintaining a balanced and adequate diet, dysphagia, weight gain, obesity or malnutrition, dehydration, and inadequate nutrient intake. A study that examined the dietary patterns of people with intellectual disabilities reported that these individuals had inadequate intake of essential nutrients such as energy, fiber, vitamin B1, folic acid, calcium, and iron, and that approximately 19.7% of these individuals were obese and 2.5% were underweight (46). Another study reported that 19.5%–50.0% of children with any disability had nutritional deficiencies, including deficiencies in vitamin D, vitamin B12, and folate in addition to biomarkers of inadequate nutrition such as low prealbumin and plasma iron levels (47). In this study of children and adolescents with disabilities, the nutrients with inadequate intakes were vitamin E, vitamin B1, folate, potassium, calcium, and iron, while the nutrients with intakes above the requirements were protein, carbohydrates, vitamins A, B2, B6, B12, and C, phosphorus, zinc, and sodium. Participants with good diet quality had higher energy and nutrient intakes and higher percentages of meeting nutrient requirements (*p* < 0.05). Inadequate nutrient intake may result from eating problems. However, it is essential that nutritionists and healthcare and community service providers target children at high risk of malnutrition (such as those with current disabilities) in already established nutrition programs to ensure effective and optimal nutrition (14).

Functional gastrointestinal disorders are very common in adolescents and children and are often associated with functional limitations, decreased quality of life, and increased healthcare costs (48). Foods can affect gastrointestinal sensitivity, motility and barrier function, as well as the gut microbiota, by modulating atypical mechanisms in the gut (49). Nutrients can have important effects on gut microbiota, modulating gut barrier function and gut motility. Nutrients are key elements of life because they can act as both triggers and therapeutics. The majority (93%) of children with functional gastrointestinal disorders identify certain foods as the cause of exacerbating gastrointestinal complaints, such as abdominal pain or diarrhea, and other foods as the cause of improving these symptoms (48). Healthy eating patterns and the Mediterranean diet, known to help reduce the risk of chronic disease, disability, and premature death, are associated with improved cognitive performance and gastrointestinal health in children and adolescents (50). A study of healthy children and adolescents aged 4–18 years from six Mediterranean countries reported that good compliance to the Mediterranean diet resulted in a significantly lower prevalence of functional gastrointestinal disorders and functional constipation (OR = 0.83, *p* < 0.001, OR = 0.89, *p* = 0.008, respectively) (49). Another study investigating the association between functional constipation and compliance with the Mediterranean diet in healthy Turkish children between the ages of 6 and 18 years reported that good compliance with the Mediterranean diet resulted in a lower risk of functional constipation. Compared with low KIDMED scores, the adjusted odds ratio for constipation was 0.71 (95% CI) for the mean score and 0.35 (95% CI) for the high KIDMED score. The odds ratio for constipation was 0.84 for a one-point increase in the KIDMED score (51). In our study, we also found that high compliance to the Mediterranean diet was linked with lower severity of gastrointestinal symptoms (*r* = −0.14, *p* < 0.001). Symptoms such as reflux, constipation, indigestion, diarrhea, and abdominal pain were associated with the adoption of a diet with a low representation of Mediterranean diet characteristics (*r* = −0.10, *p* < 0.001; *r* = −0.12, *p* < 0.001; *r* = −0.06, *p* = 0.008; *r* = −0.08, *p* = 0.001; and *r* = −0.15, *p* < 0.001). In addition, children with disabilities who consumed diets with poor Mediterranean dietary characteristics had more severe gastrointestinal symptoms than those who consumed diets with average and good Mediterranean dietary characteristics (GSRS scores: 25.8 ± 11.17 vs. 23.4 ± 9.79 and 22.6 ± 8.74, *p* < 0.001). Although the study samples vary, the results reported in the literature are similar. Studies on children and adolescents with disabilities are lacking, and this is the first study conducted on a large sample to holistically evaluate the relationship between diet and gastrointestinal health in disabled children and adolescents, warranting further studies. It is an undeniable fact that the Mediterranean dietary pattern contains elements that promote and support health. The Mediterranean diet may have a protective effect on functional gastrointestinal disorders because it contains healthy nutritional components and is characterized as a dietary pattern that preserves intestinal function and structure (52). Such a protective effect on functional gastrointestinal disorders is based on the information that nutrients from the Mediterranean diet interact with the mucosal barrier, increase the Bifidobacteria/*E. coli* ratio in the intestinal microbiota, and result in improved microbial diversity (49). The Mediterranean diet is linked with lower levels of Firmicutes and Bacteroidetes species and higher levels of fecal short-chain fatty acids (53). The high content of omega-3 polyunsaturated fatty acids and monounsaturated fatty acids make the Mediterranean diet a dietary model with anti-inflammatory and antioxidant properties (48). Because the Mediterranean diet contains high levels of fiber, monounsaturated fatty acids, polyphenols, and antioxidants, and low levels of saturated fatty acids, it may be beneficial in the improvement of functional gastrointestinal symptoms (54). However, it is argued that the Mediterranean diet may prevent dysbiosis by contributing to reduced levels of plasma inflammatory markers, including tumor interferon-γ, necrosis factor-α, and high-sensitivity C-reactive protein (48).

It is suggested that adopting a diet that is highly representative of Mediterranean diet characteristics may lead to a healthy life with improved physical activity, along with improvements in sleep quality, quality of life, and body image satisfaction (55, 56). A meta-analysis study examining quality of life in healthy children and adolescents (*n* = 6,796) reported a significant positive relationship between compliance to the Mediterranean diet and quality of life (β = 0.13–0.26) (55). In a 1-year follow-up study (*n* = 1,146), a one-unit improvement in children’s Mediterranean diet score was associated with a likelihood of improvement in overall quality of life (OR = 1.09, 95%CI) and emotional (OR = 1.09, 95%CI) and social functioning (OR = 1.13, 95%), but was not linked with physical and school functioning (57). In our study, compliance to the Mediterranean diet results in increased quality of life, including improvements in all life quality sub-dimensions (physical, emotional, social, and educational; *r* = 0.12, *p* < 0.001). A one-unit increase in the GSRS score resulted in a 14.4 times decrease in the PedsQL score, and a one-unit increase in the KIDMED score resulted in a 10.8 times increase in the PedsQL score. Health-promoting bioactive components in the Mediterranean diet are associated with a reduction in the frequency of functional constipation (58), which is associated with poor quality of life (51). The results of our study also confirm that the Mediterranean diet is associated with less severe gastrointestinal symptoms. In light of our study data, we suggest that the Mediterranean diet can increase quality of life through its potentially beneficial effects on gastrointestinal health. Furthermore, a study has shown that compliance to the Mediterranean diet mediates perceived happiness and perceived social acceptance, specifically leading to the development of a sense of being respected and accepted by peers (59). Because the children on a Mediterranean diet may have reduced depressive symptoms and improved cognitive function (60, 61), children who adopt a Mediterranean dietary pattern may have a better quality of life (57). The topicality of the issue, resulting in the availability of only a few studies, and the current limited information do not allow for a clear conclusion about disabled people. Since studies in the literature usually focus on examining the quality of life of healthy children and adolescents, there is a need to comprehensively investigate the relation between quality of life and dietary patterns in individuals with disabilities.

This is the first large sample study to evaluate the association between dietary practices and health outcomes (malnutrition, gastrointestinal symptoms, eating problems, and quality of life) in people with disabilities. However, our study has several limitations. One limitation may be that the information on gastrointestinal symptoms, quality of life, and dietary characteristics of the children and adolescents included in this study is based on parent reports. A 1-day food report for assessing energy and nutrient intakes may not reflect routine daily intake patterns. Another limitation of this study is that the level/severity of disability was not examined. Comprehensive studies of the subcomponents of disability type and severity, as well as gender-specific assessments, will help to shed light on the relationship between health outcomes and dietary patterns. However, the strength of this study is that it examines nutritional status indicators such as growth, development and malnutrition, eating problems, gastrointestinal symptoms and quality of life in three dimensions (type of disability, age, and Mediterranean diet characteristics) in a large sample size. The risk factors that lead to malnutrition and disability are multifaceted, which justifies the need for comprehensive studies to assess the relevant risks for resolving adverse nutrition-related outcomes.

## Data availability statement

The datasets presented in this article are not readily available due to restrictions (e.g., their containing information that could compromise the privacy of research participants). Requests to access the datasets should be directed to HB, handecekici@hotmail.com.

## Ethics statement

The studies involving humans were approved by Non-Interventional Clinical Research Ethics Committee of Istanbul Medipol University approved the study protocol with decision number 894 on 26 October 2022. The studies were conducted in accordance with the local legislation and institutional requirements. Written informed consent for participation in this study was provided by the participants’ legal guardians/next of kin.

## Author contributions

HB contributed to the conception and design of the study. HB, VÖ, and MP collected article data, analyzed all survey data, and contributed to all statistical analyses and interpreted data. HB and VÖ wrote the manuscript. All authors contributed to the article and approved the submitted version.
